# Microfinance investments in quality at private clinics in Uganda: a case-control study

**DOI:** 10.1186/1472-6963-7-168

**Published:** 2007-10-18

**Authors:** Eric E Seiber, Amara L Robinson

**Affiliations:** 1 Division of Health Services Management and Policy, College of Public Health, Ohio State University, 1841 Neil Avenue, Columbus, OH 43240, USA; 2 Independent Researcher, Lusaka, Zambia

## Abstract

**Background:**

Small private-sector health care providers can play an important role in meeting the developing country health care needs, but a lack of credit can prove major constraint to small-provider expansion. This study examines the potential of small, microfinance loans to strengthen the private health sector and improve access to quality preventive and curative health services in Uganda.

**Methods:**

This study estimates logistic regressions using 2,387 client exit interviews to assess the impact of microfinance loans on perceived quality and the viability and sustainability of small, private clinics.

**Results:**

The study finds perceived quality improved with loan recipients' clients being more likely to choose clinics on the basis of drug availability, fair charges, cleanliness, and confidentiality. In addition, the assessment found evidence of increased client flows, but the changes produced mixed results for sustainability with respondents being only half as likely to "always" visit a particular clinic.

**Conclusion:**

The results indicate that the microfinance program improved perceived quality at loan recipient clinics, especially as reliable drug outlets.

## Background

For several years the Ugandan government has recognized that small private-sector health care providers – including pharmacists, nurses, midwives, and doctors – can play an important role in meeting the country's health care needs, but a lack of credit has been a major constraint to small-provider expansion [1]. To address this lack of credit, the Uganda Private Providers Loan Fund was launched to provide microfinance loans and technical assistance to these small private-sector providers. Through these small loans, microfinance holds the potential to strengthen the private health sector and improve access to preventive and curative health services.

First developed by the Grameen Bank in Bangladesh, the basic micro-credit model extends small loans to impoverished individuals previously ignored by the formal banking sector. Variants of this basic microfinance model have proliferated amongst NGOs and donors, with micro-credit interventions now implemented with a multitude of goals in both industrialized and developing countries. Despite its rapid proliferation, little is known about microcredit's potential applications with small developing country healthcare providers. Multiple microfinance studies have considered the impact of microfinance on household income, female empowerment, and children's educational attainment. However, microfinance studies related to health are more limited. Previous studies have explored the domestic violence sometimes observed with some interventions with female empowerment as a goal [2], advantages of integrating the income generation dimension of microfinance with health promotion and education [3-5], and the use of existing microfinance organizations to mitigate the impact of natural disasters [6,7].

Until recently, interventions have rarely applied the microfinance model to small, private providers of health services in developing countries. There exists a general consensus that perceived quality of care is linked, at least to some degree, with the effectiveness of health care services through increased client loyalty and use of those services [8,9]. The only published study examining the impact of a microfinance program on the private sector in Uganda focused on the relationship between perceived quality of care and the utilization of RH services for a group of midwives' clinics. The study found that microfinance loans improved perceived quality of care and increased client loyalty [10]. Descriptive results for a study conducted in Indonesia indicate that midwives receiving micro-credit saw a marked increase in the number of new family planning clients within one year [11]. There are no known studies that examine the impact of micro-credit programs on services offered by private clinics with the exception of reproductive health care. This gap in the literature is particularly important in Uganda where malaria remains the number one cause of morbidity and mortality [12].

This study considers the impact of a microfinance program on clients' perceptions of quality of care and indirectly assesses the loan program's impact on the viability and sustainability of private health care providers in Uganda. The sample includes clinics operated by doctors, nurses, clinical officers, and pharmacists in addition to the midwife clinics considered by previous authors. The paper is organized in five sections. Section 1 provides background on the intervention, including the surrounding context in which the loans were rolled-out. Section 2 describes the study methodology. Sections 3 and 4 present the bivariate and multivariate results. The final section discusses the study findings and limitations.

Uganda is split up into 80 districts, which include 4 administrative areas. Kampala district, where the majority of intervention activities occurred, has the largest population at over 1.2 million people (2002 Census) and is primarily urban, containing the capital city of the same name.

With an estimated per capita income of $259 in 2003, Uganda remains one of the poorest countries in the world. Although Uganda has shown improvement in a number of health indicators, including a decreasing level of HIV prevalence, [13] others point to the country's continued need for expanded health services. This need is particularly true for women and children, the most vulnerable members of society. In 2001, the infant mortality rate in Uganda was estimated at 88 deaths per 1,000 live births, and average life expectancy at birth was 45 years. Malaria remains the most significant health problem in Uganda [14], accounting for 40% of outpatient visits [15]. Increased use of antenatal clinics by vulnerable women and the reduction of drug stock-outs, two key policy strategies under the RBM initiative, remain a formidable challenge for the public health sector in its current and future efforts to reduce malaria burden [12]. With an estimated total fertility rate of 6.9 children per women (2001) [16], increased use of antenatal clinics for RH and MCH services also remains a health and development priority for the country.

Although the Ugandan government has recognized that small private-sector health care providers could play an important role in meeting the country's health care needs for several years, a lack of credit has been a major constraint to small-provider expansion [17]. In response, the Summa Foundation, a not-for-profit investment fund created by USAID (and now operating as part of the USAID-funded Commercial Market Strategies project, or CMS), in collaboration with CMS/Uganda, launched the Uganda Private Providers Loan Fund in 2001. The fund, which focuses primarily on the Kampala district in the Central Region of Uganda, was designed to provide a package of both financing and technical assistance to small-scale private health care providers. Implementation of the fund overlapped with the government's decision to remove user fees in all community-level public facilities in the country. Although several subsequent studies on the effects of this new policy document an overall rise in outpatient utilization in public facilities after user fees are removed [18-20], they also point out the potential risk associated with abolition of fees including overcrowding, drug shortages, and overburdened staff within the public sector [18,20]. Current and past literature on the subject of equity in health care and fees-for-service has documented its inherent exclusion of the very poor. Several studies, however, have also found a willingness to pay for services, even among those with limited resources, when services are perceived to be of superior quality. [21-23].

The aim of making loan funds available was to expand and improve the services offered by private health care providers. This objective was timely as it had the potential to partially fill the gaps which developed in the public health sector due to the sudden influx of clients to those facilities. By enabling providers to invest in their practices and by offering training in business skills, the fund gives providers the opportunity to improve service quality. The underlying premise is that improved quality of care will be reflected in improved client quality-of-care perceptions – which in turn will attract more clients, increase client loyalty, and make these private sources of health care services, including malaria treatment and RH services, more sustainable. As such, the loan program had the overall goal of improving private practice viability and sustainability through improved and expanded health care services.

For this study, "viability" is defined as the feasibility or practicality of the private sector to provide quality health care related to various types of both preventive and curative services. "Sustainability" is measured in terms of client loyalty and the proportion of clients that state they always visit the clinic. The relationship between viability and sustainability is implied; the overall goal of the intervention was to make it possible for private clinics to become a feasible or practical option for clients to receive quality curative and preventive health services, including those related to RH and malaria treatment. It was assumed that increased viability of a clinic would subsequently lead to its enhanced sustainability – or its ability to attract and retain a static clientele base.

The project has made two rounds of loans. Initial loans went to 15 midwives who were recruited through the Uganda Private Midwives Association. Midwives typically work for a number of years in government service and then establish their own private clinics. Beyond reproductive health services, such clinics often provide primary care services that include administering immunizations to children and dispensing drugs to both male and female clients. An impact assessment of the first round of loans on clinic clients' perceptions of quality of care was conducted in 2002 [10].

In response to increasing demand for the program, the pool of loan recipients was expanded in 2002 to include a broader set of health providers, among them doctors, nurses, clinical officers, pharmacists, and other clinic owners. The level of available funds was increased from $175,000 to $300,000, and the program's timeframe extended for an additional two years. This study assesses the impact of the second round of Summa loans, made from October 2001 to November 2002.

Loan recipients were identified through professional associations and direct marketing carried out by the Uganda Micro-Finance Union, which has administered the fund. Just over 44 percent of second-round loan recipients were midwives and 30.5 percent were nurses. Clinical officers made up 15.4 percent, and doctors, 8.9 percent. Just over half (56.5 percent) of all borrowers resided in peri-urban areas, with the remaining loan recipients located in urban (26.5 percent) and rural (17.2 percent) settings.

Loans were disbursed to providers on a revolving basis. Average size of the loan was $920. Recipients could use loan proceeds as working capital, to purchase drugs or equipment, or to renovate or upgrade the clinic. Monitoring data indicate that the majority of loan recipients, regardless of how many times they had received a loan, planned to use a portion of the money to increase their drug stocks. The majority of first-time borrowers (82.9 percent) used a portion of their loan to purchase drug supplies, and almost half (45.3 percent) used a portion to buy equipment. More than a quarter (27.1 percent) of recipients also stated that they used part of their loan proceeds to renovate or expand their clinic. While subsequent borrowers continued to use a substantial portion of loan resources to purchase drug stocks, an increasing number also invested in equipment and clinic renovation and expansion.

It should be noted that for the purpose of this study providers in the intervention group had at least one and a maximum of two loans. Due to changing patterns in how borrowers invest loan proceeds, future evaluations of borrowers who have received more than two loans may produce different findings. The changes may have an impact on clients' perceptions, particularly as they relate to range of services offered and essential equipment.

The loan program included a five-day business skills training component conducted by the National Smallholder Business Center. Attendance at the training session was required for all providers before receiving loan funds. The training curriculum included core business-management elements such as understanding client satisfaction, along with an introduction to the family planning products sold by the CMS project.

## Methods

The study design and survey methodology used in this assessment parallel that used by Agha et al. [17]. The study employs a quasi-experimental design that uses baseline and follow-up surveys and a nonequivalent comparison group to evaluate the impact of the loans on the outcomes of interest.

This study considers 29 private clinics, of which 22 received a Summa loan (the intervention group) and seven did not (the comparison group). Twenty-three clinics – 18 intervention and five comparison – were located in the Kampala district; the remainder were located in either the Mukono or Wakiso districts surrounding Kampala. The majority of both intervention and control clinics were located in peri-urban areas (68% and 60%, respectively) and averaged three employees. CMS developed the survey instrument and supervised the training of all interviewers. Each interviewer remained at the same clinic throughout the data collection period.

See Additional File 1 for the number of exit interviews completed for each study group during the baseline and follow-up surveys.

Baseline data were collected during October 2001, and the follow-up survey was administered in October and November 2002. Exit interviews of all clients leaving the clinics were conducted over a five-day period. Survey acceptance was high, with only a few clients declining to participate.

The questionnaire was designed to collect data on socio-demographic characteristics of clients (including age, gender, marital status, educational attainment level); reason for visit; and level of client satisfaction. The survey instrument was pre-tested using several clinics in Kampala, and appropriate changes were made. Measurement of intervention clinics' viability and, subsequently, their ability to sustain the provision of curative and preventive health services was based on three dimensions of health care service: a) expansion of services, b) improvements in client perceptions of quality of care, and c) sustainability of services. The specific indicators measuring changes in these three dimensions are listed below:

▪ Expansion of services: Availability of drugs, range of services;

▪ Improvements of services/quality of care: Availability of drugs, fair charges, cleanliness, good handling of clients, privacy, accessibility, good physical outlook, range of services, essential services;

▪ Sustainability of services: Always visit clinic.

In addition, clinic "viability" or its feasibility or practicality to provide health care services was measured in terms of equity of care, or a clinic's ability to reach clients regardless of differences in background characteristics such as gender and socioeconomic status.

Descriptive analyses of the client exit interviews compare the profiles of clients at baseline and follow-up, as well as changes in client response between baseline and follow-up. The bivariate results establish which quality dimensions are most important to Ugandan clients and illustrate changes in the quality indicators over time. All bivariate analysis used Pearson chi-squared and Wald tests to test for significant differences between clinic groups and over time. Each clinic is treated as its own stratum in calculating the test statistic.

Multivariate analysis was then required in order to attribute the changes over time observed at the bivariate level while controlling for any observed differences in clientele background characteristics. These adjusted odds ratios test the hypothesis that the indicator has changed between baseline and follow-up, after controlling for differences in respondent demographics between the two survey rounds. Separate logistic regressions for loan and comparison clinics estimate the model:

*Perceived_Quality *= *α *+ *β*_1_*Follow_up *+ *β*_2_*Age *+ *β*_3_*Female *+ *β*_4_*Some_education *+ *β*_5_*Secondary_education *+ *β*_6_*Expenditures_per_capita*

Where:

Perceived_Quality = the quality indicator of interest

Follow_up = 1 if interview conducted in the follow-up round (time trend)

Age = age of the respondent

Female = 1 if respondent is female

Some_education = 1 if respondent has some schooling, but has not finished secondary school

Secondary_education = 1 if respondent finished secondary school

Exenditures_per_capita = food and rent expenditures per household member in thousands of Ugandan Schillings.

The coefficient β_1 _in each model, tests the significance of the time trend for the comparison and intervention clinics, independent of the demographic characteristics in the sample. The model treated each clinic as a separate stratum when calculating standard errors for the estimates. A significant time trend indicates that perceived quality has changed, independent of respondent demographics, but the time trend is insufficient to attribute the change to the intervention.

A final model estimated from the pooled data from all clinics and survey rounds determined the net impact of the project. Logistic regression for all respondents estimated the final model:

*Perceived_Quality *= *α *+ *β*_1_Loan_Group*Follow_up + *β*_2_*Loan_Group *+ *β*_3_*Follow_up *+ *β*_4_*Age *+ *β*_5_*Female *+ *β*_6_*Some_education *+ *β*_7_*Secondary_education *+ *β*_8_*Expenditures_per_capita*

This final model includes all variables from the time trend model, but also adds two new variables. Since loans cannot be issued randomly to clinics, a dummy variable indicating clinic group controls for differences in quality between loan and comparison groups at baseline. Second, a time trend clinic group dummy variable assigns impact by testing if perceived quality changed more at loan clinics than comparison clinics. The model treated each clinic as a separate stratum when calculating standard errors for the estimates.

## Results

Tables 1 through 5 display the results of the bivariate analysis. Since the comparison clinics provide the reference point for measuring program impact, the first table examines the intervention and comparison clinics at baseline to assess the validity of making comparisons between these two groups. Due to data limitations, the descriptive analyses can make only a rough approximation of changes in client volume over the study period.

**Table 1:** Socio-demographic characteristics of clients at baseline (n = 1270)

	**Intervention (n = 967)**	**Comparison (n = 303)**	**Significant Difference**
	
**Mean age**	28.0	27.3	-
			
**Gender (percent distribution)**			**-**

Male	41	37	
Female	59	63	
			
**Marital status (percent distribution)**			**

Never married	35	36	
Married	59	61	
Other	7	3	
			
**Education (percent distribution)**			**

None, some primary	16	18	
Completed primary	12	19	
Some secondary	37	35	
Completed secondary or higher	34	28	
			
**Usual sources of treatment (percent distribution)**			***

Public hospital/clinic	13	8	
Private hospital/clinic	82	80	
Pharmacy	1	1	
Drug shop	3	11	
			
**Lives in neighborhood (percent distribution**			-

Yes	78	81	
No	22	19	
			
**Mean number of individuals in household**	5.0	4.5	***

			
**Mean household expenditures **(Schillings)			

On rent and food	136,619	117,870	***
Per capita	34,918	30,548	***
			
**Respondent currently uses FP (percent distribution)**	47	42	-

*** Significant at the α = 0.01 confidence level

** Significant at the α = 0.05 confidence level

* Significant at the α = 0.10 confidence level

**Table 2:** Clients' reason for visiting the clinic rather than another clinic, at baseline and follow-up (percent)

	**Intervention**			**Comparison**		
	
**Reasons for visit today**	**Baseline **(n = 884)	**Follow-up **(n = 856)	**Significant Difference**	**Baseline **(n = 306)	**Follow-up **(n = 261)	**Significant Difference**
Availability of drugs	31	41	***	55	31	***
Fair charges	21	37	***	39	42	-
Cleanliness	9	19	***	19	25	*
Good handling of clients	50	52	-	47	52	-
Privacy	6	18	***	17	13	*
Accessibility	57	49	***	65	62	-
Good physical outlook	2	4	***	2	12	***
Range of services	13	9	**	9	11	-
Has essential equipment	3	5	***	3	7	**
						
**Always visit the clinic**	43	37	***	41	47	-

*** Significant at the α = 0.01 confidence level

** Significant at the α = 0.05 confidence level

* Significant at the α = 0.10 confidence level

**Table 3:** Preventive and curative reasons for visiting clinics at baseline and follow-up (percent)

	**Intervention**			**Comparison**		
	
**Preventive Reason**	**Baseline **(n = 902)	**Follow-up **(n = 856)	**Significant Difference**	**Baseline **(n = 318)	**Follow-up **(n = 261)	**Significant Difference**
Family Planning^†^	5	6	-	8	6	-
MCH (includes FP)^†^	17	18	-	19	18	-
						
**Curative Reason**						

Malaria treatment^†^	37	40	-	42	30	***
Other reason (curative, drugs, and misc.)	50	43	***	39	52	***

^†^Note: MCH/FP and Malaria not exclusive

*** Significant at the α = 0.01 confidence level

** Significant at the α = 0.05 confidence level

* Significant at the α = 0.10 confidence level

**Table 4:** Reason for visiting the clinic for midwives and other providers receiving loans (percent)

	**Midwives Receiving Loans**			**Other Providers Receiving Loans**		
	
**Reasons for visit today**	**Baseline **(n = 299)	**Follow-up **(n = 351)	**Significant Difference**	**Baseline **(n = 585)	**Follow-up **(n = 505)	**Significant Difference**
Availability of drugs	29	41	***	33	41	***
Fair charges	21	40	***	22	35	***
Cleanliness	10	17	***	9	20	***
Good handling of clients	53	47	-	49	56	**
Privacy	5	18	***	6	17	***
Easily accessible	51	60	**	61	42	***
Good physical outlook	4	4	-	1	5	***
Range of Services	11	15	-	14	6	***
Has Essential Equipment	3	2	-	3	8	***
						
**Always visit the clinic**	47	42	-	40	33	**

*** Significant at the α = 0.01 confidence level

** Significant at the α = 0.05 confidence level

* Significant at the α = 0.10 confidence level

**Table 5:** Preventive and curative reasons for visiting clinics for Midwives and Other Providers Receiving Loans (percent)

	Midwives Receiving Loans			Other Providers Receiving Loans		
	
**Preventive Reason**	**Baseline **(n = 304)	**Follow-up **(n = 351)	**Significant Difference**	**Baseline **(n = 598)	**Follow-up **(n = 505)	**Significant Difference**
Family planning^†^	6	8	-	4	4	-
MCH (includes FP)^†^	23	20	-	14	16	-
						
**Curative Reason**						

Malaria treatment^†^	36	42	*	37	39	-
Other reason (curative, drugs, and misc.)	47	39	**	52	46	**

^†^Note: MCH/FP and Malaria not exclusive

*** Significant at the α = 0.01 confidence level

** Significant at the α = 0.05 confidence level

* Significant at the α = 0.10 confidence level

Table 1 shows the distribution of respondents in the intervention and comparison groups by selected demographic and household characteristics. These client characteristics indicate that there are modest, but statistically significant differences in client SES between the comparison and follow-up clinics. Individuals using intervention clinics proved slightly more likely to be male, finished secondary school, have a larger family, and have higher expenditures per capita in their household. Respondents in both groups had a mean age of about 28 years. There were no significant differences in the proportion of men and women interviewed in the two groups, with women making up less than two-thirds of respondents at both intervention and comparison clinics.

The marital status of respondents showed a minor significant difference between groups, with the significance arising from the four-point difference in those reporting Other Marital Status (7 percent versus 3 percent). Respondents visiting intervention clinics were significantly more likely to have higher levels of education than clients interviewed at comparison clinics. Only 28 percent of respondents in the comparison group reported completing at least a secondary-level education, compared to 34 percent of respondents surveyed at intervention clinics. Clients visiting clinics receiving loans reported larger households than those visiting comparison clinics (5 versus 4.5 persons per household), and enjoyed higher household socioeconomic status.

The results in Table 2 indicate that before controlling for the differences in client SES, intervention clinics showed broader improvement in the quality indicators than the comparison clinics. Perceived quality significantly increased for six indicators at intervention clinics compared to improvements in only two indicators at comparison clinics. Both groups experienced statistically significant declines for two indicators each.

Since most credit recipients stated they would use their loans to purchase drugs, client perception of the availability of drugs is of particular interest. Over the study period, the proportion of clients who stated that they visited an intervention facility instead of an alternate clinic due to the availability of drugs increased from 31 percent at baseline to 41 percent at follow-up. This same indicator showed a significant decrease for comparison clinics, dropping from 55 percent at baseline to 31 percent at follow-up.

In addition to drug availability, more intervention clinic clients cited perceived fairness of charges, cleanliness, privacy, physical appearance, and the presence of essential equipment as their reason for choosing the clinic at follow-up. The percentage of clients stating they always visit this facility decreased over the study period.

Table 3 examines the preventive and curative reasons for clinic visit, before controlling for client characteristics. Between baseline and follow-up, neither group of clinics experienced any significant change in clients seeking FP or MCH preventive visits. All significant changes manifested within the curative reasons. Almost 25 percent of clients at both intervention and comparison clinics sought preventive care for the surveyed visit; only 5 to 8 percent of all clinic visits were for family planning services.

In contrast to the lack of change in preventive services, the mix of curative and malaria visits shifted for both intervention and control clinics. At the bivariate level, intervention clinics experienced a modest increase in the proportion of patients citing malarial treatment (including malarial drugs) as their primary reason for choosing that clinic, pushing down the percentage citing other curative visits as the factor driving their choice of clinic. Clients at intervention clinics who stated they visited the clinic for malaria treatment increased from 37 to 40 percent, while those citing "other reasons" decreased significantly from 50 to 43 percent between baseline and follow-up, respectively. In contrast, comparison clinics saw a significant increase in the proportion of clients seeking other curative reasons services while visits specifically for malarial treatment visits declined.

While Tables 2 and 3 considered the pooled changes for the indicators across all providers receiving loans, previously published research indicates that microfinance loans can increase perceived quality at clinics operated by midwives [17]. The limited number of clinics precludes a statistical testing of the differences at midwife clinics and clinics operated by physicians and clinical officers, but Tables 4 and 5 provide weak evidence that loans have had similar effects across all providers.

Table 4 reveals a striking similarity in results for midwives and other providers receiving loans. Each group showed similar starting values and near identical changes for the categories "Availability of Drugs," "Fair Charges," "Cleanliness," and "Privacy." Although results diverged for several of the other indicators, it is not possible to indicate why. Three divergent trends do, however, merit highlighting. First, midwives saw more clients choosing their clinics due to accessibility (increasing from 51 percent to 60 percent), while other providers saw a large drop (from 61 to 42 percent) in their clients citing accessibility as a reason for their visit. Second, while the percentage of clients visiting midwives due to the presence of essential equipment remained largely unchanged, other providers saw a modest increase (from 3 to 8 percent) of clients citing essential equipment as a reason for their visit. Finally, while the percentage of respondents who always visit the clinic decreased for both provider groups, midwives started with higher client loyalty and saw an insignificant drop. Other providers started from a lower level of loyalty and suffered a larger, significant drop.

Table 5 repeats the patterns of Table 4, with both provider groups generally displaying similar changes over the study period. For preventive visits, neither provider type saw a significant change in the percentage of clients visiting for family planning or MCH visits (although midwives do provide a substantially higher percentage of client visits for preventive reasons). The percentage of visits for malaria treatment did change for midwives, increasing from 36 percent to 42 percent, while malaria treatment visits for other providers stayed relatively flat, moving from 37 to 39 percent.

The small, but significant, differences in client demographics at loan and comparison clinics confound the degree to which the changes observed in Tables 2 and 3 are due to the loan program. Tables 6 and 7 take the same indicators examined previously and test for a net change between survey rounds, controlling for client demographics and time invariant characteristics of intervention and comparison clinics. Net program impact was determined by the interaction model described in the Methodology section. The odds ratio for net impact was estimated by pooling all interviews from both intervention and comparison clinics, and indicates whether clients' perceptions of quality at intervention clinics changed relative to comparison clinic perceptions over the study period.

**Table 6:** Adjusted odds ratios indicating changes in reasons for visiting the clinic (n = 2,278)

**Reasons for visit today**	**Intervention **(n = 1,729)		**Comparison **(n = 549)		**Net effect **(n = 2,278)	
Availability of drugs	1.55	***	0.37	***	4.21	***
Fair charges	2.16	***	1.11	-	1.96	***
Cleanliness	2.26	***	1.46	*	1.60	**
Good handling of clients	1.08	-	1.22	-	0.90	-
Privacy	3.63	***	0.66	*	5.17	***
Accessibility	0.73	***	0.87	-	0.83	-
Good physical outlook	2.21	***	6.17	***	0.38	*
Range of services	0.71	**	1.28	-	0.57	*
Has essential equipment	1.98	***	2.75	**	0.90	-
						
**Always visit the clinic**	0.73	***	1.39	*	0.51	***

*** Significant at the α = 0.01 confidence level

** Significant at the α = 0.05 confidence level

* Significant at the α = 0.10 confidence level

**Table 7:** Adjusted odds ratios indicating changes in preventive and curative reasons for clinic visit (n = 2,307)

**Preventive Reason**	**Intervention **(n = 1,747)		**Comparison **(n = 560)		**Net effect **(n = 2,307)	
Family planning	1.05	-	0.63	-	1.54	-
MCH (includes FP)	0.97	-	0.94	-	1.10	-
						
**Curative Reason**						

Malaria treatment	1.17	*	0.63	***	1.86	***
Other reason (curative, drugs, and misc.)	0.77	***	1.71	***	0.45	***

*** Significant at the α = 0.01 confidence level

** Significant at the α = 0.05 confidence level

* Significant at the α = 0.10 confidence level

These pooled estimates in Table 6 demonstrate substantial changes between baseline and follow-up for intervention clinics compared to the control clinics. Of particular interest is the significant increase in clients visiting intervention clinics due to the perceived availability of drugs. Clients at intervention clinics were just over 1.5 times more likely in the follow-up than the baseline round to report availability of drugs as the reason for their current visit. Comparison clinic clients were about one-third as likely to state this reason. The observed difference resulted in a large net positive program impact, with clients at intervention clinics proving 4.2 times more likely than clients at comparison clinics to cite availability of drugs as the reason for clinic choice.

The same trend emerged for the indicator measuring perceived privacy of client visit. Respondents visiting loan clinics were over three times more likely at follow-up to state that they had chosen that particular clinic because of a confidential environment. In contrast, clients at comparison clinics became *less *likely to visit current clinic because of perceived privacy. These findings also produced a positive net program impact, with respondents surveyed at intervention clinics over five times more likely to cite privacy as the main reason for their visit.

The program also improved the likelihood of visiting an intervention clinic due to fair charges for services (just over 2 times more likely) and general cleanliness of the facility (about 2.5 times more likely). Again, these positive impact results represent the relative improvement of intervention clinics compared to comparison clinics between baseline and follow-up surveys.

Although clients at both comparison and intervention clinics proved more likely to cite the facility's good physical appearance as a reason for their visit, this trend resulted in a net negative program effect. In this case, both groups showed improvement, but the comparison group showed an unusual 10 percent increase (from 2 to 12 percent) in clients identifying appearance as a reason for their visit. Finally, the net negative program effect on clients' visit due to the perceived range of services derived from both a modest drop at intervention clinics and an insignificant increase at comparison clinics.

Over the study period, loan clinic clients proved less likely to report that they "always visit this clinic." Although this result seems to suggest that loan clinics experienced a decline in client loyalty, further examination indicates that it stems from more clients reporting that they "sometimes" use the clinic. Because the data consist of two cross sections, we cannot explicitly track an individual client's clinic preferences. This result may be due, in part, to the concurrent abolishment of user fees at public facilities that created a "crowding-out" effect at some private facilities due to a sudden increase in new clients ("sometimes") visiting intervention clinics. With two cross sections, a broadening of the clinic's client base with more casual users would cause the percentage of clients who responded "always visit the clinic" to decline.

While the data cannot fully answer if this decline is attributed to lower client loyalty or to a broadening of the client base, the data collection format does allow a rough estimate of weekly utilization (since each sample includes all clients for a particular week). If the decline in loyalty comes from new clients, then utilization must have increased. In the year between the baseline and intervention surveys, loan clinics did see the total number of visits during data collection week at baseline and follow-up increase by five clients per week; a 12 percent average increase over comparison clinics. While this finding does suggest that client flows have increased, the result applies only to the week of the sample and cannot be extrapolated to an annual estimate.

Table 7 examines the multivariate and net impact results for the preventive and/or curative reasons for client visit. As observed, clients at loan clinics were almost 1.2 times more likely to visit the facility for malaria-associated care at follow-up. Clients of comparison clinics, however, were less than two-thirds as likely to be at the clinic at follow-up for malaria treatment. These trends resulted in a net positive program effect, with a loan clinic client being nearly two times more likely to be seeking malaria treatment at follow-up. Program impact was also associated with the observed decrease in clinic visits for "other reasons." This decrease reflects a compositional shift in visits away from the general "other reason" to the more specific "malaria treatment" category.

In Table 8, loan clinics enjoyed higher client loyalty. For both survey rounds, clients at intervention clinics were 1.45 times more likely to always choose that clinic. However at follow-up, clients were .45 times less likely to state that they always visit a loan clinic. As discussed in relation to Table 6, this apparent decrease in client loyalty includes the effect of mixing in an average of 12 percent more new clients at loan clinics at follow-up.

**Table 8:** Adjusted odds ratios indicating factors associated with always visiting the clinic (n = 2,273)

**Variable**	**Odds Ratio**	
Loan Group at follow-up	0.45	***
Loan Group	1.45	***
Follow-up	1.36	*
Age	1.01	***
Female	1.36	***
Married	1.22	**
Expenditure per capita^1^	1.01	-
		
**Education^2^**		

Less than Secondary	0.89	-
Finished Secondary	0.53	***
		
**Reason for Visit**		

Availability of drugs	1.48	***
Fair charges	1.72	***
Cleanliness	1.50	***
Good handling of clients	2.31	***
Privacy	0.77	*
Accessibility	1.22	**
Good physical outlook	1.32	-
Range of Services	0.89	-
Has Essential Equipment	0.74	-

^1 ^Thousands of Ugandan Schillings

^2 ^No Education is the base category.

*** Significant at the α = 0.01 confidence level

** Significant at the α = 0.05 confidence level

* Significant at the α = 0.10 confidence level

Of particular programmatic interest are the associations between clients' reasons for their visit and continued loyalty. Respondents who cited availability of drugs as a reason for the clinic visit were 1.5 times more likely to always choose that loan clinic. Similarly, clients who perceived value in services received (fair charges) were 1.7 times more likely to frequent the same clinic. Perceived cleanliness of the loan clinics and the feeling that they were treated well were also positively associated with client loyalty and subsequent visits. Interestingly, visiting a clinic due to a perceived broad of range services and essential equipment were not important predictors for return to that facility.

## Discussion

The main objective of the microfinance program has been to improve three dimensions of health care service provision and, subsequently, the viability and sustainability of the small-scale practices of private health care providers who received one or more loans. The data suggest that clients at intervention clinics did perceive improvements in the expansion of services and several indicators of quality of care. There were mixed results, however, with regard to the intervention's overall goal of improved viability and future sustainability of loan clinics to provide both preventive and curative health care services.

The strongest finding was that the program improved sustainability at loan clinics through the enhanced and increased provision of curative services, namely the consistent availability of drugs. Several studies on the effects of the abolition of cost-sharing in 2001 at public facilities cite drug shortages at public facilities as one of the major short-comings of the new policy [18,19,24]. Furthermore, this same literature found that while utilization at public facilities rose dramatically after user fees were removed, there did not appear to be a simultaneous decrease in clients at private facilities that continued to charge for services; it appears that, when able, people are willing to pay for services when the quality is perceived superior to an alternative free option [18]. The fact that intervention clients were more likely to be of an upper SES strata and visit a clinic for malaria treatment (i.e., able to pay for "superior" or, in this case, available malaria drugs) support this finding.

Despite the improvements in these elements of perceived quality, the changes led to mixed results for client loyalty. At follow-up, loan clinic respondents were only half as likely to "always visit that clinic." Closer examination of the data suggests, however, that this change may stem from more clients reporting that they "sometimes" use the clinic. As the program hoped, loan clinics did seem to attract new clients – while the data allowed only a partial answer to this question, data collection did provide a rough weekly utilization estimate (since all clients in a given week were interviewed). The comparison between baseline and follow-up surveys showed that loan clinics saw an average increase of five clients per week (a 12% average increase) over comparison clinics. While this finding indicates that client flows did increase for the interview week, the result cannot be extrapolated to an annual estimate.

The decline in the proportion of clients reporting that they "always" use the loan clinics may be due to the abolishment of user fees at government clinics that coincided with the provision of loans to these clinics. Further, the decline in client loyalty at clinics receiving loans could be explained by new clients who encountered declining quality at government clinics due to a sudden influx of patients and are now considering loan clinics as an alternative source of care. With this interpretation, new clients are disproportionately choosing loan clinics for malaria related services compared to "other reasons" for the comparison clinics.

Although anticipated, it is important to note that "viability" of intervention clinics, or the practicality of loan clinics to provide quality health care services, was limited to clients who could afford to pay. On the other hand, it can be argued that the largest improvement in loan clinic sustainability came through strengthened revenue from increased drug sales. Despite perceptions about the range of services and the presence of essential equipment remaining largely unchanged over the study period, respondents were four times as likely to cite drug availability in the follow-up survey as the reason for their loan clinic choice. This finding coincides with follow-up loan clinic clients being half as likely to cite drug availability as an area needing improvement.

Caution should be used however when discussing "quality" in terms of increased provision of drugs, particularly those related to the treatment of malaria. Drug resistance due, at least in part, to the over-prescription of the most widely used malarial drugs has been cited as one of the greatest challenges facing Africa in the fight against malaria [25]. At least one other African study examining the effect of improvements in drug availability and quality of care in the private and public sector found that the two were not necessarily congruent [26]. Quality of care in the provision of drugs not only demands their availability, but also proper diagnostics, prescription practices, and correct utilization. Finally, the limited records kept by providers prevented the monitoring of specific drug stocks over the study period. Future research will be needed to examine the relationship between increased supply and the proper provision of drugs in the Ugandan context.

The loans found less success in increasing preventive visits. The results found no change at loan clinics for the percentage of respondents seeking preventive or family planning services. Although the loans showed no impact on family planning utilization, it is nevertheless important to remember that they have strengthened the supply of reproductive health (RH) services available from the private sector in Uganda. In case of a future setback in public-sector resources, these private providers will remain a viable source of RH services to Ugandan women.

Finally, the evaluation design and the available data produce several limitations for the findings. The evaluation utilized a random sample of clients from each clinic, treating each clinic as its own stratum. This design implies that these results can be generalized to the population of clients utilizing these 29 clinics but not directly to other private Ugandan clinics. Second, limitations in the clinic financial and performance data required the evaluation to focus on client perceptions of clinic performance. These two limitations highlight the opportunity for future research to develop low cost instruments appropriate for collecting performance measures from small clinics. Future studies utilizing these low cost, provider specific instruments could address gaps in the existing literature on the role of private clinics in the health systems of developing countries.

## Conclusion

The public health sector in Uganda is expected to face financing constraints for the foreseeable future while demand for its services continues to increase [14]. This paper shows that microfinance interventions can strengthen private providers and enable them to cover important gaps in the public sector. In the case of these Ugandan providers, microcredit produced a range of quality improvements, including strengthening the perception of these providers as reliable pharmaceutical providers, and generating an increased demand for malaria treatment services. For environments that lack Uganda's high malaria burden, further research will be necessary to determine the response of the private sector healthcare providers to microfinance interventions.

## Competing interests

Uganda Private Providers Loan Fund described in this paper was created as a collaboration between the Summa Foundation, a not-for-profit investment fund established by the United States Agency for International Development (USAID) (and now operating as part of the USAID-funded Commercial Market Strategies project, or CMS), in collaboration with CMS/Uganda. The authors performed this study under contract with the Commercial Market Strategies project.

## Authors' contributions

ES led the design of the study and data analysis, and helped draft the manuscript. AR contributed to the data analysis and led the writing of the manuscript. All authors read and approved the final manuscript.

## Pre-publication history

The pre-publication history for this paper can be accessed here:



## Supplementary Material

Additional file 1 Additional file 1 Click here for file
