# Supplementary material for: Microfinance investments in quality at private clinics in Uganda: a case-control study
Source: BMC Health Serv Res. 2007 Oct 18;7:168. doi: 10.1186/1472-6963-7-168 (PMC2244795; doi:10.1186/1472-6963-7-168)
Supplement: Additional file 1 [file 1472-6963-7-168-S1.doc]

##### Baseline Follow-up

Intervention clinics 951 856

Comparison clinics 319 261
